# Perioperative Immunonutrition in Surgical Cancer Patients: A Summary of a Decade of Research

**DOI:** 10.1007/s00268-013-2323-z

**Published:** 2013-11-01

**Authors:** Stanislaw Klek, Piotr Szybinski, Kinga Szczepanek

**Affiliations:** General and Oncological Surgery Unit, Stanley Dudrick’s Memorial Hospital, 15 Tyniecka Street, 32-050 Skawina, Poland

## Abstract

**Background:**

Immunonutrition is assumed to enhance immune system function. In surgical patients, it is supposed to reduce postoperative complications. However, results of recent clinical trials have been puzzling and have not supported this theory.

**Aim:**

The aim of our study was to evaluate the value of enteral and parenteral postoperative immunonutrition.

**Methods:**

After initial evaluation of 969 patients, the intent-to-treat analysis included 776 patients (female 407, male 466, mean age 61.1 years) undergoing gastric or pancreatic resections between 2001 and 2009. All patients were randomly assigned after surgery to one of the following groups: standard enteral nutrition (SEN), immunomodulating enteral nutrition (IMEN), standard parenteral nutrition (SPN), or immunomodulating parenteral nutrition (IMPN). All malnourished patients received preoperative parenteral nutrition. Number and type of postoperative complications, length of hospitalization (length of stay [LOS]), and vital organ function were assessed.

**Results:**

No statistically significant differences were observed in well-nourished patients, during either enteral or parenteral intervention, independent of the type of intervention (standard or immunomodulating). However, analysis of the malnourished group revealed the positive impact of enteral immunonutrition on reduction of postoperative complications (28.3 vs. 39.2 %, respectively; *p* = 0.043) and LOS (17.1 and 13.1 days, respectively; *p* < 0.05) compared with a standard enteral diet. The cross-analysis of SEN, IMEN, SPN, and IMPN was insignificant.

**Conclusions:**

The type of postoperative nutrition was of no importance in well-nourished patients. However, in malnourished patients, enteral immunonutrition helped to improve treatment outcome. These findings suggest its use as a method of choice during the postoperative period.

## Introduction

Only a few factors may influence results of surgery to such an extent as malnutrition. It complicates wound healing, increases the rate of postoperative infections, and lengthens hospital stay. These outcomes are consequences of the destruction of immune function, amplifying the response to stress and organ dysfunction [1].

Nutritional therapy has been used in the postoperative period for over 100 years, since Kausch administered intravenous glucose solution to help his patient’s recovery [2]. However, the history of nutritional support as we know it nowadays began with the invention of parenteral nutrition by Dudrick et al. [3]. Intravenous admixtures proved effective not only in maintaining health status, but also in safeguarding growth. This medical approach irreversibly changed the perception of perioperative care. However, Buzby et al. [4] and the Veteran Affairs Trial [5] highlighted the consequences of hyperalimentation in well-nourished patients, indicating a reduction in complications of up to 20 %, but only in malnourished patients. These studies clarified the perspective of perioperative care and switched a proportion of nutritional intervention to the more physiological, less expensive, and safer enteral feeding [4, 5].

It took another decade to set the criteria for the selection of the proper feeding route. Nowadays, leading scientific societies, ASPEN (American Society for Parenteral and Enteral Nutrition) and ESPEN (European Society for Clinical Nutrition and Metabolism), agree that enteral nutrition should be used as the method of choice in perioperative treatment [6, 7]. Further studies concentrated on pharmaconutrition: formulas that can be used not only to deliver basic nutrients, but also to influence vital organs and systems to improve the outcome of therapies. One form of pharmaconutrition, aiming at improvement of immune function, was called immunomodulating or immunostimulating. These diets, both parenteral and enteral, included amino acids (arginine and glutamine), lipids (omega-3 fatty acids), micronutrients (vitamins C and E), and nucleotides. Soon after their implementation, some authors observed their encouraging influence on the outcome of surgery, which raised new hope for surgical patients [8–10]. Controversy soon began, and the actual value of immunomodulating formulas for surgical and critically ill patients was examined. The positive effects of immunodiets observed in experimental models were often denied by clinical results, far more important for clinicists [11]. In contradiction to Braga et al. [8]. or Gianotti et al. [9], Senkal et al. [10]. and Lobo et al. [11]. revealed that enteral immunodiets bore no advantage over standard enteral nutrition (SEN) when a peptide diet was used; other authors noticed analogous results. Furthermore, most research performed in well-nourished individuals failed to demonstrate the quantifiable efficacy of immunomodulating diets [11–13].

These outcomes were difficult to match because of the heterogeneity of study populations, study designs, sample quantities, and systematic approaches.

To completely address these uncertainties and to verify the value of immunonutrition in surgical patients, randomized well-designed trials were conducted in a tertiary surgical center between 2001 and 2009. Results of these trials were partially published in medical journals, but have never been presented as a summary [12–15].

## Methods

### Study design

We conducted a randomized, not blinded (due to obvious differences in enteral and parenteral routes), controlled study in order to evaluate the impact of enteral and parenteral immunonutrition on postoperative complications in surgical patients. The study was conducted in the tertiary surgical center—the 1st Department of General and Oncology Surgery, Jagiellonian University School of Medicine, Cracow, Poland—and was performed between January 2001 and December 2009.

The research was planned to test the hypothesis that immunonutrition would decrease the occurrence of surgical and non-surgical complications after gastrointestinal (GI) surgery. The secondary objectives included evaluation of effects of nutritional intervention on morbidity and mortality, length of hospital stay (LOS), and vital organ function.

### Patient characteristics and inclusion/exclusion criteria

A total of 969 patients were initially assessed for participation in the study; 96 patients were unable to meet inclusion criteria and were excluded after initial assessment. The intent-to-treat (ITT) analysis of 776 patients (female 407, male 466, mean age 61.1 years) who underwent gastrectomy (subtotal or total resection) or pancreatoduodenectomy (subtotal or total) with lymph node excision were enrolled in the trial. The inclusion criteria included good overall status (Karnofsky >80, Eastern Cooperative Oncology Group [ECOG] grade 0 or 1); the absence of cancer dissemination or severe associated diseases (cardiac, pulmonary, renal, liver failure, chronic obstructive pulmonary disease [COPD], coronary aortic bypass graft, etc.); no history of known aversions or intolerance to analyzed substances. Patients with metastatic or unresectable cancer, who were pregnant or in poor general health (Karnofsky <80, ECOG > 1), with recent history of severe heart, lung, kidney, or liver failure, with history of allergies or drug intolerance were excluded. Malnutrition was defined as either of the following: unintentional weight loss of at least 10–15 % within 3–6 months before admission or body mass index (BMI) <18 kg/m^2^. Respective groups of patients were comparable with each other in terms of sex, age, type of surgery, BMI, weight loss, serum albumin, and total lymphocyte count (TLC) on admission and blood transfusion.

### Randomization and allocation of patients

**Fig. 1:**
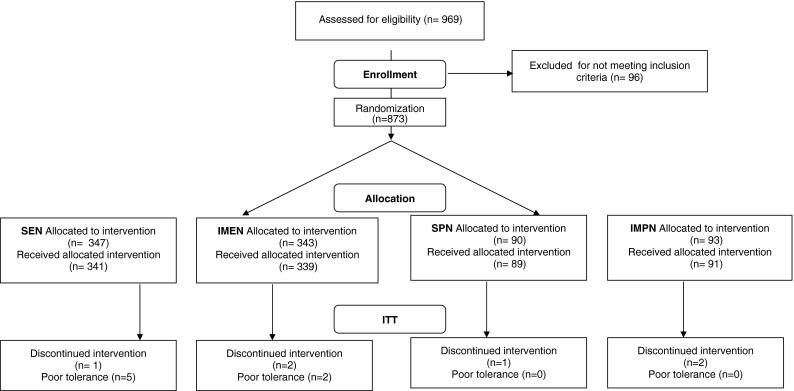
CONSORT diagram showing the flow of participants through each stage of the trial. *CONSORT* Consolidated Standards of Reporting Trials, *IMEN* immunomodulating enteral nutrition, *IMPN* immunomodulating parenteral nutrition, *ITT* intent-to-treat population, *SEN* standard enteral nutrition, *SPN* standard parenteral nutrition

After tumor resection, individuals who met the eligibility norms were intraoperatively allocated to either of four groups using sealed envelopes containing computer-generated distribution numbers. A 2 × 2 factorial scheme was used with the subsequent groups: SEN, immunomodulating enteral nutrition (IMEN), standard parenteral nutrition (SPN), and immunomodulating parenteral nutrition (IMPN) in two parts of the research. In the third part, in which enteral intervention was assessed, patients were randomized into SEN or IMEN groups. The CONSORT (Consolidated Standards of Reporting Trials) diagram (Fig. 1) shows the flow of participants through the study.

### Clinical management

In contrast with well-nourished patients, who underwent surgery without preoperative nutritional support, all malnourished patients received intravenous nutrition the fortnight before surgery. Protein and energy demands were calculated using the nitrogen to body weight (b.w.) ratio (0.15 g N/kg b.w.) and the non-protein energy proportion (*Q* = 150 kcal/g N). The 10 % amino acid solutions, 10–40 % glucose, and 10–20 % lipid emulsions, trace elements (Aminoplasmal, Glucose and Lipofundin MCT/LCT B and Tracutil, B Braun, Germany), vitamins (Cernevit, Baxter, USA), and electrolyte solutions were used to prepare all-in-one bags at the pharmacy. The central intravenous catheter was implanted in the subclavian or jugular vein before the onset of therapy. The tip location was confirmed by chest X-ray. The same type of intravenous admixture as given preoperatively was provided to each patient during the postoperative period up to postoperative day 7, or longer in case of complications.

The selection of parenteral instead of enteral feeding during the preoperative period, which has been endorsed for many years, was the consequence of the absence of those guidelines at the time of study preparation (2001) and the wide acceptance of that kind of perioperative approach in local surgical units (Polish national standards).

During surgery (gastric or pancreatic resection), an enteral feeding tube (Flocare Nutricia Ltd., 140 cm length) was inserted into the first intestinal loop, 15–20 cm below the nethermost anastomosis. The surgical team included at least two skilled general and oncological surgeons, and the anesthesiology team comprised four people.

Preceding surgery, BMI, weight loss, full blood count with TLC, albumin and pre-albumin concentration, liver and kidney tests were assessed.

On postoperative day 1, 3, and 8, the following assessments were made: full blood count with TLC, serum albumin and pre-albumin concentrations, liver and renal function, quantity of diet administered, and tolerance. Assessments were performed by physicians and nurses.

The energy and protein requirements during the postoperative period were calculated using the same method as during the preoperative intervention. Enteral feeding was started 6 h after the procedure, with 5 % glucose solution at the rate of 20 ml per h for the first 12 h, followed by infusion of Peptisorb (SEN group; Nutricia Ltd., Poland) or Reconvan^®^ (IMEN group; Fresenius Kabi, Poland) at the rate of 20 ml/h on day 1, 50 ml/h on day 2, 75 ml/h on day 3, and 100 ml/h thereafter. The whole intervention lasted 1 week. Diet ingredients are presented in Table 1. Infusion devices were used to administerr the diet for 20–22 h, with a 2–4 h rest period. An oligopeptide, isocaloric diet was selected as a control because of previous high tolerance in the small intestine. Infusion pumps were used to guarantee volume and speed control.

**Table 1:** Composition of enteral diets (per 100 ml/100 kcal)

Ingredient	Peptisorb (standard diet)	Stresson (immunodiet)	Reconvan^®^ (immunodiet)
Energy (kcal)	100	125	100
Amino acids (g)	4.0	7.5	5.5
Carbohydrates (g)	17.6	14.5	18.0
Polysaccharides (g)	14.6	13.3	13.3
Sugars (g)	1.7	0.7	0.7
Lactose (g)	0.1	<0.025	0.15
Total fat (g)	1.7	4.1	3.3
Saturated (g)	1.0	2.6	3.3
MCT (g)	0.8	1.5	1.2
Fibre (g)	0	0.1	0
Electrolytes			
Sodium mg (mmol)	100 (4.3)	134	138
Potassium mg (mmol)	150 (3.8)	263	207
Chloride mg (mmol)	125 (3.5)	139	141
Calcium mg (mmol)	80 (2.0)	67	80 (2.0)
Phosphorus (mg)	72 (2.3)	67	60
Magnesium (mg)	23 (0.9)	28	25
Iron (mg)	1.6	1.0	1.33
Zinc (mg)	1.2	1.0	1.2
Copper (mcg)	180	338	133
Manganese (mg)	0.33	0.63	0.63
Fluoride (mg)	0.10	0.13	0.27
Molybdenum (mcg)	10.0	13	10.0
Selenium (mcg)	5.7	14	6.7
Chromium (mcg)	6.7	8.9	6.7
Iodine (mcg)	13	17	13.3
Vitamins			
Vitamin A (mcg RE)	82	91	70
Vitamin D (mcg)	0.70	0.88	0.88
Vitamin E (mg α-TE)	1.3	1.3	1.0
Vitamin K (mcg)	5.3	6.6	6.7
Thiamine (mg)	0.15	0.19	0.2
Riboflavin (mg)	0.16	0.31	0.16
Niacin (mg NE)	1.8	2.3	1.6
Pantothenic acid (mg)	0.53	0.66	0.47
Vitamin B6 (mg)	0.17	0.39	0.16
Folate (mcg)	27	50	27
Vitamin B12 (mcg)	0.21	0.4	0.27
Biotin (mcg)	4.0	5.0	5.0
Vitamin C (mg)	10.0	25	6.7
Choline (mg)	37	46	26.7
Taurine (mg)	10	13	13
Glutamine (g/l)	–	10.1	10.2
Arginine (g/l)	–	7.2	6.7

### Primary objective (primary endpoint)

The primary objective of this study was to assess the influence of immunomodulating nutrition on postoperative complications in surgical patients. The ratio of postoperative complications was nominated as the primary outcome measure, with the hypothesis that the routine use of immunodiets in the postoperative period reduces the number of infectious and surgical complications. Definitions of complications are presented in Table 2.

**Table 2:** Definitions of complications

Complication	Definition
Wound infection	Purulent exudate in the wound with positive bacterial culture
Abdominal abscess	Collection of pus confirmed by percutaneous drainage or at reoperation
Pneumonia	Clinical signs of pneumonia and/or radiographic evidence (both required to diagnose) or positive culture of tracheal aspirate or blood or brushing
Urinary tract infection	Clinical symptoms and the presence of bacteria in urine (>100,000 colony-forming units/ml)
Bacteremia	Positive blood culture
Infection of venous catheter	Local signs of inflammation, and/or the isolation of pathogen organisms in culture
Sepsis	Fever >38 °C or hypotension (<90 mm Hg systolic BP) or oliguria (<20 ml/h) along with positive blood culture
Wound dehiscence	Any dehiscence of the fascia >3 cm
Bleeding	Necessity of blood transfusion (≥2 U)
Anastomotic leak	Positive dye-swallow or contrast-swallow test
Respiratory tract failure	Presence of dyspnoea and respiratory rate >35 breaths/min or PaO_2_ <70 mm Hg
Circulatory insufficiency	Unstable BP requiring use of extra fluids or cardiac stimulants
Renal failure	Necessity of haemodialysis
Hepatic dysfunction	Increased serum bilirubin (2–3 times above baseline) or hepatic enzyme level (3–4 times above baseline)
Pancreatic fistula	Drain output of any measurable volume of fluid on or after postoperative day 3 with an amylase content greater than 3 times the serum amylase activity
Delayed gastric emptying	Necessity for nasogastric suction for ≥8 days after surgery

*BP* blood pressure

### Secondary objectives (secondary endpoints)

The secondary objectives included LOS, immune system function (clinical observations and TLC), assessment of liver and renal function, and treatment compliance. Furthermore, operational time, intra-operative blood loss, blood transfusions, and the necessity for re-operation were recorded. Post-operative mortality was defined as any fatal outcome within 31 days after hospitalization. The length of postoperative stay was number of days from the date of operation until the date of discharge. Albumin solutions were not used as standard treatment.

### Sample size and statistical analysis

The sample size was calculated using SamplePower™, version 16–19 (SPSS Inc., Chicago, IL, USA). A general estimation was made for each study: the total rate of complications after upper GI surgery described by previous studies was approximately 40 %. To detect a 50 % reduction triggered by immune enteral nutrition, more than 82 patients should be randomized to each of the two related arms (alpha = 0.05 two-sided, power = 0.80). We assumed a drop-out rate of 15 %; therefore, 200 patients were needed. The figures were investigated on an ITT basis using SPSS v.14 software. The differences in proportions amid groups were assessed using the Chi squared test, and Yates correction was implemented if any of the probable incidences were <5. Continuous data were studied using the Mann–Whitney U test. Differences at *p* < 0.05 were considered statistically significant.

### Ethics and consent

The Ethics Committee of Jagiellonian University sanctioned the study (KBET/91/L/2004). It was not possible to obtain approval in 2001, as the Ethics Committee did not participate in such activities beforehand. Patients were registered by one of two investigators (SK, KS). Written approval was acquired from each participant before acceptance. The study was carried out following the universal ethical endorsements stated in the Helsinki Declaration and was recorded in the Clinical Trials Database (NCT00576940).

### Role of sources of funding

The study was performed with no outside sponsorship.

## Results

Participants were adequately matched for age, sex, weight loss, BMI, type of operation (stomach/pancreas resection), TLC, and serum albumin (indicators of nutritional state). The number of patients who were operated on for gastric and pancreatic neoplasm, as well as patients within both groups, was comparable. The ratio of resection types (sub/total excision) did not differ between groups.

During preoperative intravenous feeding in malnourished patients, each study group received a comparable level of energy, proteins, and micronutrients.

### Postoperative follow-up

There were no noteworthy dissimilarities between the enteral groups in the tube feeding delivery, either in the malnourished or the well-nourished group. For the purpose of this research, the observation was completed on postoperative day 8, after a full 7 days of enteral feeding, but the mean length of intervention in the SEN and IMEN group was 8.4 (±1.2) days and 8.6 (±1.4) days. The mean interval of intravenous nutrition was 7.9 (0.8) days for SPN and 8.1 (1.0) days for IMPN.

Compliance was similar amid malnourished patient groups, protocol violation due to the full dose of diet not being delivered was the reason for premature cessation in eight patients (SEN-1, IMEN-2), which accounted for <1 % of all patients. The planned delivery reached 80 % of those originally prescribed. The average quantity of blood units was 1.7 in SEN and 1.6 in IMEN, which was not significant (*p* = 0.42).

Detailed analyses of postoperative complications are presented in Tables 3, 4, 5 and 6.

**Table 3:** Postoperative complications among study groups (part 1: 2 × 2 randomization in malnourished patients)

Variable	SEN and SPN (*N* = 84)	IMEN and IMPN (*N* = 83)	OR (95 % CI)	*p* value	PN (*N* = 83)	EN (*N* = 84)	OR (95 % CI)	*p* value
Rate of infectious complications	23 (27)	20 (24)	0.842 (0.420–1.687)	0.627	19 (23)	24 (29)	1.347 (0.671–2.706)	0.401
Overall morbidity	33 (39)	25 (30)	0.666 (0.351–1.265)	0.214	29 (35)	29 (35)	0.982 (0.519–1.857)	0.995
Morbidity (30 days post-surgery)	37 (41)	28 (33)	0.655 (0.353–1.345)	0.216	32 (38)	32 (38)	0.983 (0.499–1.878)	0.991
Mortality	3 (4)	0 (0)	0.882 (0.473–1.643)	0.692	1 (1)	2 (2)	1.078 (0.579–2.008)	0.812
Mortality (30 days post-surgery)	4 (5)	1 (1)	0.789 (0.541–1.742)	0.691	2 (2)	3 (3)	1.112 (0.611–1.997)	0.828
Hospital stay, days [median (IQR)]	9 (9–14)	9 (9–12)	–	0.835	9 (9–13)	9 (9–12)	–	0.415

Data are presented as *N* (%) unless otherwise indicated

*EN* enteral nutrition, *IMEN* immunomodulating enteral nutrition, *IMPN* immunomodulating parenteral nutrition, *IQR* interquartile range, *OR* odds ratio, *PN* parenteral nutrition, *SEN* standard enteral nutrition, *SPN* standard parenteral nutrition

**Table 4:** Postoperative complications among study groups (part 2: enteral nutrition in malnourished patients)

Type of complication	SEN (*N* = 153)	IMEN (*N* = 152)	*p* value^*^
Infectious complications	60 (39.22)	43 (28.29)	0.04366
Pneumonia	45 (29.41)	33 (21.71)	0.12322
Urinary tract infection	15 (9.80)	11 (7.24)	0.42213
Surgical wound infection	27 (17.65)	12 (7.89)	0.01077
Intra-abdominal abscess	10 (6.54)	5 (3.29)	0.18988
Bacteremia	11 (7.19)	2 (1.32)	0.01112
Sepsis	2 (1.31)	4 (2.63)	0.40498
Other complications			
Wound dehiscence	8 (5.23)	2 (1.32)	0.05502
Pancreatic fistula	10 (6.54)	4 (2.63)	0.10329
Intestinal fistula	8 (5.23)	4 (2.63)	0.24340
Duodenal fistula	1 (0.65)	2 (1.32)	0.55793
Biliary fistula	2 (1.31)	3 (1.97)	0.64672
Abdominal fluid collection	2 (1.31)	3 (1.97)	0.64672
Delayed gastric emptying	13 (8.50)	8 (5.26)	0.26479
Acute pancreatitis	1 (0.65)	2 (1.32)	0.55793
Intestinal obstruction	2 (1.31)	2 (1.32)	0.99473
Peritonitis	1 (0.65)	1 (0.66)	0.99629
Pulmonary embolism	2 (1.31)	1 (0.66)	0.56563
Heart failure	3 (1.96)	2 (1.32)	0.65738
Respiratory failure	7 (4.58)	5 (3.29)	0.56362
Liver failure	1 (0.65)	0 (0.00)	0.31810
Renal failure	0 (0.00)	3 (1.97)	0.08075
Neurological	1 (0.65)	1 (0.66)	0.99629
Peripheral veins thrombosis	2 (1.31)	1 (0.66)	0.56563
GI bleeding	1 (0.65)	1 (0.66)	0.99629
Abdominal bleeding	2 (1.31)	2 (1.32)	0.99473
Mortality	9 (5.88)	2 (1.32)	0.03247
Overall morbidity	72 (47.06)	51 (33.55)	0.01621

All data are presented as *N* (%) unless otherwise indicated

*GI* gastrointestinal, *IMEN* immunomodulating enteral nutrition, *SEN* standard enteral nutrition

**Table 5:** Postoperative complications among study groups (part 3: 2 × 2 randomization in well-nourished patients)

Variable	Standard nutrition (*N* = 102)	Immunonutrition (*N* = 103)	OR (95 % CI)	*p* value	PN (*N* = 100)	EN (*N* = 105)	OR (95 % CI)	*p* value
Rate of infectious complications	28 (27)	25 (24)	0.81 (0.43–1.50)	0.498	25 (25)	28 (27)	1.14 (0.61–2.14)	0.672
Overall morbidity	36 (35)	37 (36)	1.08 (0.60–1.93)	0.804	35 (35)	38 (36)	0.85 (0.48–1.50)	0.577
Mortality	2 (2)	2 (2)	0.99 (0.14–7.17)	0.992	2 (2)	2 (2)	0.95 (0.13–6.89)	0.960
Hospital stay (days, mean)	12.8	12.5	–0.32 (–1.62 to 2.26)	0.746	12.9	12.4	–0.43 (–2.31 to 1.46)	0.656

Data are presented as *N* (%) unless otherwise indicated

*EN* enteral nutrition, *OR* odds ratio, *PN* parenteral nutrition

**Table 6:** Postoperative complications among study groups (part 2: enteral nutrition in well-nourished patients)

Type of complication	SEN (*N* = 91)	IMEN (*N* = 92)	*p* value
Infectious complications			
Pneumonia	15 (16.4)	13 (14.1)	>0.05
Urinary tract infection	1 (1.1)	2 (2.1)	>0.05
Surgical wound infection	5 (5.5)	4 (4.2)	>0.05
Abscess formation	2 (2.2)	2 (2.1)	>0.05
Surgical complications			
Evisceration	0	1 (1.1)	>0.05
Pancreatic fistula	1 (1.1)	0	>0.05
Duodenal fistula	1 (1.1)	1 (1.1)	>0.05
Jejunal fistula	2 (2.2)	1 (1.1)	>0.05
Biliary fistula	0	1 (1.1)	>0.05
Surgical complications overall	4 (4.4)	4 (4.4)	>0.05
General complications			
Pulmonary thrombosis	0	0	>0.05
Myocardial infarct	0	1 (1.1)	>0.05
Peripheral vein thrombosis	0	0	>0.05
Neurological complications	0	0	>0.05
Fatal outcome	1 (1.1)	1 (1.1)	>0.05
Complications overall (patients)	21 (23.1)	23 (25.2)	>0.05
Uncomplicated postoperative period (patients)	70 (76.9)	69 (75)	>0.05

Data are presented as *N* (%) unless otherwise indicated

*IMEN* immunomodulating enteral nutrition, *SEN* standard enteral nutrition

The LOS differed between the two study groups and extended up to 17.1 days (standard deviation [SD] 12.2) in the SEN group and 13.1 days (SD 13.8) in the IMEN group (*p* = 0.006) in malnourished patients. In the same group, there were significant dissimilarities in infectious complications, which occurred in 60 patients (39.2 %) in the SEN group and 43 (28.3 %) in the IMEN group (*p* = 0.04). Differences were also detected in morbidity (47.1 vs 33.5 %; *p* = 0.01) and mortality (5.9 vs 1.3 %; *p* = 0.03).

In well-nourished patients, the median LOS was 12.4 (SD 5.9) days in the SEN group and 12.9 (SD 8.0) days in the IMEN group (*p* = 0.42). Complications were detected in 21 patients (23.1 %) in the SEN group and 23 (25.2 %) in the IMEN group (*p* > 0.05). Four (4.4 %) patients in the SEN group and four (4.4 %) in the IMEN group had surgical complications (*p* > 0.05).

Blood transfusions were necessary in 12 well-nourished patients in the SEN group and 11 in the IMEN group; the median numbers of transfused units were 2.5 in SEN and 2 in IMEN (interquartile range [IQR] 1–3.5 and 1–5, respectively).

In these patients, LOS was similar in both groups: 12.4 (SD 5.9) days in the SEN and 12.9 (SD 8.0) days in the IMEN (*p* = 0.42) groups. Complications were noted in 21 (23.1 %) patients in the SEN and 23 (25.2 %) in the IMEN groups. Infective complications were detected in 23 patients in the SEN group and 21 in the IMEN group.

Well-nourished patients administered parenteral and enteral nutrition had a morbidity rate of 36 %; the occurrence of specific complications was similar among all groups (Table 4). Infectious complications were detected in 28 of 102 patients on standard diets and in 25 of 103 patients receiving immuno-formula (odds ratio [OR] 0.81; 95 % CI 0.43–1.50) (Table 5). Furthermore, there were no dissimilarities amid infectious complications between those receiving enteral nutrition (25 of 100 patients) and those receiving parenteral formulas (28 of 105, OR 1.14, 95 % CI 0.61–2.14). Neither the immunodiet nor enteral nutrition affected secondary outcome measures, including morbidity, mortality, and LOS.

Serum pre-albumin, albumin, and TLC levels were secondary endpoints. The first two were used to assess visceral protein synthesis and the restoration of nutritional status. However, the study did not indicate any differences among groups, as demonstrated in Table 6. Significant differences were found only in TLC on postoperative day 3, when the mean number of lymphocytes was higher in the IMEN than the SEN group (*p* = 0.011), which was not reflected in postoperative clinical course.

No differences were recorded in hepatic and renal function. These were assessed by clinical status and laboratory tests. The mean concentrations of aspartate aminotransferase (SGOT), alanine aminotransferase (SGTP), blood urea nitrogen (BUN), and creatinine concentrations did not differ.

## Discussion

Modern surgery tries to reduce the rate of postoperative complications by concentrating more on technical aspects and less on metabolic aspects. The latter probably represents the only hope for improvement in the discipline, as there are not many opportunities for further improvement from the technical point of view. It is a holistic approach that gives hope for improvement. Nowadays, the multimodal approach to perioperative care should include analgesia, physiotherapy, aseptics, antiseptics, anticoagulants, infusion therapy, nutritional support, and many other therapeutic options. Nutritional intervention matters the most, as the worsening of nutritional status has been acknowledged as a crucial factor influencing surgical outcomes [1].

The place of pre- and postoperative nutrition is no longer in question; particularly since it has been confirmed that, in severely malnourished individuals scheduled for major GI surgery, it was advantageous to postpone surgery for up to 10–14 days and to administer nutritional support, preferably with enteral diets [6]. From the surgical point of view, it was not enough to stop there; for over 10 years the focus has been on understanding immunologic and inflammatory responses, so as to enhance host defense mechanisms and improve clinical course. These activities led to the idea of immunonutrition, a type of pharmaconutrition that has been described as nutritional intervention, not only able to provide essential nutrients to maintain basic organ functions, but also to augment the immune system [16]. The use of various biochemical agents, such as non-essential (glutamine, arginine) or sulfur-containing amino acids, omega-3-polyunsaturated fatty acids, nucleotides, and anti-oxidants (free radical scavengers), administered simultaneously, in few, some or alone, was assumed to alter the host immune response [16, 17].

Several clinical trials and meta-analyses have described the beneficial effect of perioperative administration of an enteral or parenteral formula containing immune-ingredients on the outcome of surgery, independent of nutritional state [18–20]. Benefits included reduction of postoperative complications and shortening of LOS in both surgical and critically ill patients [20–25]. These results were independent of age, particularly when patients received admixtures pre-operatively [26–28]. Beneficial effects were also detected at the sub-clinical level: immunonutrition led to an increase in immune function due to an increase in TLC, CD4 levels, immunoglobulin (Ig)-G levels, and decrease in interleukin (IL)-6 concentrations, and the inversion of the correlation between IL-6 and prealbumin concentrations after surgery [8, 9, 21]. Senkal et al. [10]. observed beneficial effects of immunotherapy and even better cost effectiveness during the late phase of recovery (defined as the time period after postoperative day 5); at that time, the effect of immunodiets was incontrovertible.

However, criticism came with some studies on immunonutrition that were not able to demonstrate reduction of either overall mortality or morbidity, failed to prove benefits of immunonutrition, and indicated no reduction in complications or LOS [12, 28–31]. Some authors observed only a reduction in infectious complications without any cost-effectiveness benefits, particularly in well-nourished patients [30, 31]. Likewise, only some trials confirmed that such formulas might lower the ratio of infectious complications, and a few even suggested that immunonutrition could increase the risk of death in the critically ill [32].

There are several explanations for these inconsistencies. Most important is the question of study group: numerous studies in which immune-intervention presented no clinical effect were undertaken in well-nourished patients, while trials indicating a decrease in complications involved moderately or severely malnourished individuals [33–35]. It was obvious that any type of surgical intervention in malnourished patients would be beneficial, therefore results from malnourished patients in mixed study populations would overbalance the lack of positive results in well-nourished patients, as proven by the fresh meta-analysis of 13 randomized, controlled trials including 1,269 patients that demonstrated that perioperative immunonutrition in GI surgical patients reduced rates of postoperative infection (OR 0.41, 95 % CI 0.30–0.54), shortened LOS, and increased several markers of immune function [4]. However, nearly all of these trials comprised patients with and without malnutrition, and the percentage of malnourished patients in some of them reached nearly 60 [6–8].

Furthermore, the heterogeneity of definitions used in clinical studies to define simple concepts was confusing. Kudsk et al. [33] and Lobo et al. [11]. specified these inconsistencies: various definitions of malnutrition and co-morbidities, imprecise timing, and route of administration; various durations of therapy; uncontrolled execution of the nutritional intervention; and occurrence of nutrition support-related complications [7, 36].

Another point is that many patients undergoing upper GI surgery are at fairly low risk of fatal outcome after elective procedures, in contrast to critically ill patients. Therefore, ingredients such as arginine, which may be helpful in surgical patients, can be unsafe in the latter, because the high arginine content drives nitric oxide assembly [33].

Hence, the configuration of enteral diet plays a vital role. Studies in which Impact^®^ (Novartis) was the tested substance showed benefits, even in well-nourished patients, as validated by Daly et al. [34] and Waitzberg et al. [27, 35]. It is important to bear in mind that Impact^®^ has a specific composition: the amount of arginine in Impact^®^ is twice as low as that in, for instance, Reconvan^®^ (Fresenius Kabi). Furthermore, it contains no glutamine, while the concentration of this amino acid is quite significant in Reconvan^®^ and other enteral immunodiets. Finally, in contrast with other diets, it also contains nucleotides.

The timing of the intervention represents another perilous issue. During the postoperative period, the patient goes through contrasting stages: systemic inflammatory response (SIRS) and compensatory anti-inflammatory response (CARS), which hamper maintenance of homeostasis [9]. Thus, the same substance delivered pre-operatively may have a useful effect since inflammatory processes during these periods are dissimilar. It is also easier to accomplish a nutritional plan before than after surgery [7].

The current study was designed to confirm the hypothesis that the treatment of choice, preoperative enteral nutrition enhanced with immune ingredients, can reduce the rate of infectious complications in surgical cancer patients, who represent one of the most challenging groups of patients. Study groups were perfectly homogenous in terms of baseline characteristics, type and timing of intervention, and nutritional status. We observed that, in well-nourished patients, it was the nutritional intervention itself, not its characteristics, that mattered the most. The median postoperative LOS was 12.4 (SD 5.9) days in the SEN group and 12.9 (SD 8.0) days in the IMEN group (*p* = 0.42). Infectious complications were observed in 21 patients (23.1 %) in the SEN group and 23 (25.2 %) in the IMEN group (*p* > 0.05). The rest of the study participants did not differ.

Conversely, in malnourished patients undergoing GI surgery, it was possible to demonstrate the positive effect of IMEN on treatment outcome. The most significant clinical parameters varied markedly in favor of immunonutrition. LOS was shorter: 17.1 days in the SEN group versus 13.1 in the IMEN group; the overall morbidity (47.1 vs. 33.5 %), mortality (5.9 vs. 1.3 %), and infectious complications (39.2 in SEN vs. 28.3 % in IMEN) were reduced.

Neither diet influenced hepatic and renal function, visceral protein production, or immune system recovery. The difference observed in TLC on day 3 was too slight to have been considered clinically important.

These results reinforced the value of immunonutrition confirmed previously in the preoperative period by Braga et al. [8. and Gianotti et al. [9, 20], and in the postoperative period by Zheng et al. [21] and Heyland et al. [22]. They also support the concept that the administration of arginine- and nucleotide-rich, glutamine-free enteral diets could be advantageous in malnourished and even in some well-nourished patients. However, well-nourished patients are unlikely to gain from this management during the postoperative period.
